# Developing the implicit association test to uncover hidden preferences for sustainable drainage systems

**DOI:** 10.1098/rsta.2019.0207

**Published:** 2020-02-17

**Authors:** Emily O'Donnell, Shaun Maskrey, Glyn Everett, Jessica Lamond

**Affiliations:** 1 School of Geography, University of Nottingham, Nottingham NG7 2RD, UK; 2 Environment Agency, King's Meadow House, Reading RG1 8DQ, UK; 3 Centre for Architecture and Built Environment Research, University of the West of England, Bristol BS16 1QY, UK

**Keywords:** sustainable drainage systems, public perceptions, blue–green infrastructure, implicit association tests, greenspace, feeling thermometers

## Abstract

Understanding public perceptions of Sustainable Drainage Systems (SuDS) is critical for addressing barriers to their implementation. Perceptions are typically evaluated using explicit measures (e.g. questionnaires) that are subject to biases and may not fully capture attitudes towards SuDS. A novel image-based application of the Implicit Association Test is developed to investigate unconscious perceptions of SuDS in public greenspace and combined with explicit tests to evaluate perceptions of greenspace with and without SuDS, focusing on a sample population in Newcastle-upon-Tyne. Greenspace with or without SuDS is perceived positively by the sample population. Overall, respondents implicitly and explicitly prefer greenspace without SuDS and perceive greenspace without SuDS as more attractive, tidier and safer. The wide distribution of scores for SuDS, nonetheless, suggests a range of opinions and illustrates the complex nature of preferences for the use of greenspace. That the strongly negative explicit scores were not reflected in the implicit tests may suggest that explicit attitudes towards tidiness and safety may not be deep-rooted and are subject to social bias. Combined explicit and implicit tests may help us to understand any disconnect between expressed positive attitudes to natural spaces and behaviours around them and inform SuDS design to increase public acceptance.

This article is part of the theme issue ‘Urban flood resilience’.

## 1. Introduction

Urban flood risk and water management is gradually evolving from sole reliance on traditional ‘grey’ infrastructure (e.g. flood barriers, subsurface piped drainage) to employing a combination of grey and Blue–Green infrastructure (BGI) and Sustainable Drainage Systems (SuDS) to help meet the challenges of climate change and urban growth [1,2]. SuDS features, including retention basins, swales, rain gardens and filter trenches [3], are widely used to treat and attenuate runoff and provide multiple co-benefits to the environment and society, including climate change adaptation, improved wildlife and biodiversity, health and well-being improvements, neighbourhood amenities and aesthetic value [4]. Unlike traditional infrastructure, SuDS are often visible ‘living’ interventions that require support from residents and local government to be effectively implemented, maintained and sustained [5], yet perceptions of residents living close to SuDS are poorly understood [6]. Understanding public perception of SuDS in public greenspace is a critical step in addressing challenges to their implementation and improving awareness and support [5,7].

Perceptions of blue–green approaches to manage urban water and flood risk are typically evaluated by explicit, or self-report, measures, such as questionnaires and Likert scale tests that illustrate stated preferences [8–11]. Using structured questionnaires, Bastien *et al.* [6] found that residents value SuDS ponds for the wildlife that they attract, yet display concerns over health and safety risks and the presence of litter. Risks associated with open water bodies were highlighted by residents with children and pets. The aesthetic value of blue–green SuDS, however, makes them desirable places to live near to [12]. Several studies reiterate general explicit preferences for blue–green approaches to flood risk management over grey infrastructure with regard to aesthetics [11,13,14] yet highlight negative perceptions of ‘mess’ and littering, often influenced by site-specific physical characteristics including plant choice and maintenance regime [5]. To date, there has been little investigation to unravel these conflicting attitudes or understand why expressed positive attitudes towards BGI do not necessarily translate to supportive behaviours and actions around them. This could include taking part in positive behaviours such as monitoring or maintenance as part of local stewardship, or refraining from negative behaviours such as littering [15]. The general ‘liking’ of BGI contrasts with a general lack of explicit engagement with project proposals and consultations, and reluctance to provide funding for BGI schemes that are often experienced in practice [16].

Explicit measures to determine public perceptions of SuDS assume that an individual knows and can articulate their beliefs [17] and has an internalized concept of SuDS that they consciously base their attitudes on. The limited public awareness of the functionality of SuDS features [5,13] suggests that people may not hold strong opinions of drainage features in the public realm and view them as just ‘greenspace', as illustrated in an interview on bioswales in Portland, Oregon, USA; ‘You educated me—I didn't know it's for the water. I just thought it's pretty, and it looks nice for the neighbourhood.' [5]. Derkzen *et al.* [10] found that specific benefits of green infrastructure that are perceived as having a more direct effect on people's health and well-being (e.g. recreation and reduction of air pollutants) were better understood than less direct benefits such as temperature regulation and carbon sequestration. Similarly, the importance of green infrastructure in enhancing health and well-being, and to facilitate contact with nature, is highly valued [18].

Increasing scrutiny of the assumption that individuals have the ability to report their attitudes explicitly, as required in traditional self-reporting methods, has led to the development of new methods that reside outside of conscious awareness and control [19]. In this paper, we explore how novel tests that reveal subconscious attitudes (implicit preferences) may supplement traditional explicit measures to further enhance understanding of perceptions that may influence public attitudes towards SuDS in public greenspace. Implicit measures, such as the Implicit Association Test (IAT), are not dependent on participants' awareness of the existence or strength of associations being assessed [20] and can highlight attitudes that people were not aware that they had. The spontaneous nature of the measurement of implicit attitudes removes many of the external influences associated with measuring explicit attitudes [21] and negates issues of social desirability bias, self-enhancement bias and self-ignorance bias common with explicit tests [17].

The IAT reveals implicit attitudes by measuring the strengths of concept-attribute associations. Response times to different pairings of target-concept and attribute stimuli presented on a computer screen are compared [22]. For each trial, participants match stimuli (e.g. Rose or Beetle) with the appropriate concept (e.g. Flower or Insect) as quickly as possible. Two concepts are then combined (Flower and Good; Insect and Bad). Implicit attitudes are calculated as the difference between the average response times for compatible trials (Flower and Good; Insect and Bad) and incompatible trials (Flower and Bad; Insect and Good). The IAT has been used in investigations of perceived environmental hazards, such as nuclear power [23] and climate change [24], and to evaluate implicit connectedness with nature [17,25,26]. IATs have yet to be used to identify attitudes and preferences related to flood risk and water management, hence the insights provided by this approach could assist with the creation of blue–green spaces that provide both the required drainage function and a public space that is valued and supported by local residents.

In this paper, we investigate perceptions of SuDS in public greenspace and compare explicit and implicit attitudes, measured by a feeling thermometer and IAT, respectively, of residents in Newcastle-upon-Tyne, UK. We build on a trial version of the IAT conducted in Bristol [27] and further explore whether proximity to SuDS influences public perceptions by comparing responses from residents living (a) adjacent to established SuDS features, and (b) near to large areas of recreational greenspace.

## 2. Field site and participants

Newcastle-upon-Tyne is situated on the northwestern bank of the River Tyne in northeast England. As Lead Local Flood Authority, Newcastle City Council is responsible for managing flood risk across the city and is a statutory consultee for surface water management issues in planning applications. They work in partnership with other Flood Risk Management Authorities, including Northumbrian Water and the Environment Agency (EA), to manage fluvial and pluvial flood risk. The promotion of multifunctional blue–green space to meet environmental, social and economic needs of communities, in addition to managing water and flood risk, is embedded in policy and practice. For instance, new development will prioritize the use of SuDS given the multifunctional benefits to water quality, green space, habitat enhancement and flood risk and water management (Policy CS17, Core Strategy and Urban Core Plan, [28]). The 2016 Newcastle City Strategic Surface Water Management Plan identifies significant opportunities for retro-fitting SuDS in Newcastle city centre along five key ‘Blue/Green’ corridors, including the greening of residential streets in dense residential areas (e.g. Wingrove), natural flood management and water attenuation on existing large areas of greenspace (e.g. Town Moor) and improvements to conveyance routes and drainage with swales (e.g. St. James Boulevard) and surface water outfalls to the River Tyne [29].

Earlier research into stated preferences of practitioners in Newcastle found that lack of knowledge, education and awareness of BGI was a key barrier to gaining support from the public [7]. Conversely, a postal survey to determine opinions of SuDS ponds in one of the Newcastle Great Park (NGP) development cells [30] found a strong awareness among residents; 61% were aware of the ponds' purpose before receiving the survey and 73% said they understood the role of the ponds. Reducing flood risk was rated as important or very important by 94% of respondents, followed by a contribution to local wildlife corridors (93%) and improving aesthetics (86%). Understanding of SuDS functionality was found to be linked with ‘liking’ the ponds and willingness to engage [30], suggesting that familiarity may have an effect on perceptions, and thus, providing the motivation to test this further in this study.

**Figure 1.**
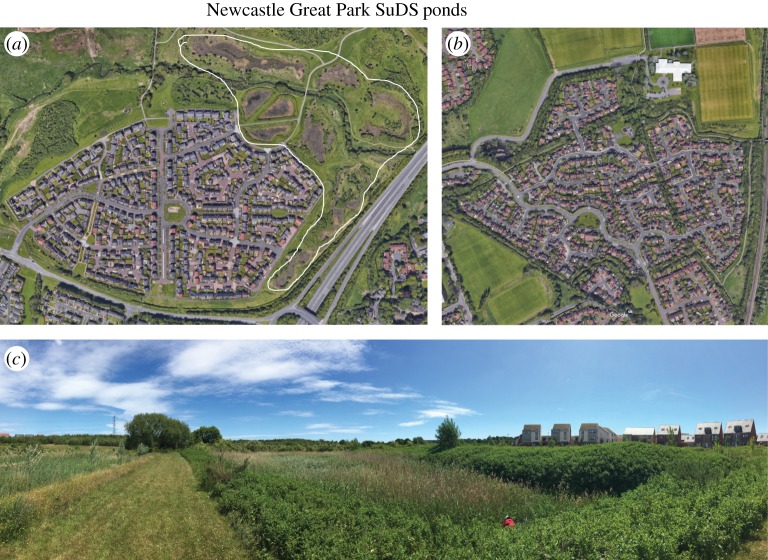
Sampling sites for Newcastle surveys. (*a*) Newcastle Great Park (NGP; proximal to several SuDS ponds that are inside the white line), (*b*) residential area in Benton proximal to greenspace including the Northumbria University Coach Lane campus and (*c*) looking northeast over two of the NGP SuDS ponds. (Online version in colour.)

Two sites in Newcastle were surveyed for this study. To reduce the potential influence of socio-economic factors, we selected areas within two 2011 Census Lower Layer Super Output Areas (LSOA) that had different types of BGI and were comparable for several socio-economic indicators, including percentages of different ethnic groups, residents' qualifications, levels of unemployment, full and part-time employment and one-family households. A residential area within NGP (figure 1*a*,*c*, Newcastle-upon-Tyne LSOA 001E) was selected as the SuDS site. Stormwater retention ponds were integrated into the NGP development to meet EA requirements. Surveyed properties were located within 500 m of SuDS features, with some directly overlooking them. A residential area in Benton near to large areas of recreational greenspace, including the Northumbria University Coach Lane campus, was selected as the no-SuDS sampling site (figure 1*b*, Newcastle-upon-Tyne LSOA 007B).

## 3. Material and Methods

### (a) Resident surveys

**Figure 2.**
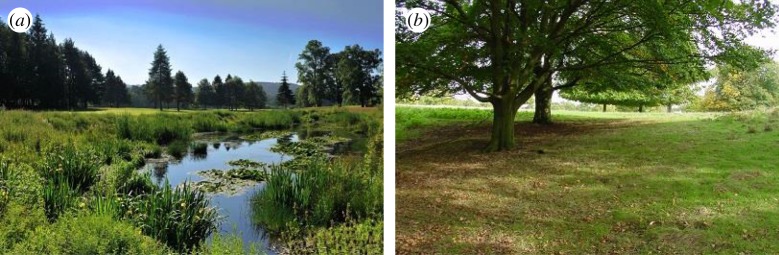
Example of images used in the Implicit Association Test (IAT). (*a*) Greenspace with SuDS and (*b*) greenspace without SuDS. (Online version in colour.)

A market research company was employed to survey residents in the two locations. Participants read a participant information sheet and granted consent prior to completing the tests. Participants were first shown 15 photographs of public greenspace with SuDS (electronic supplementary material, S1; examples of the images used in each category are shown in figure 2) and asked to spend 2 min studying the images to familiarize themselves with the types of features. They then completed the SuDS feeling thermometer (detailed in §3*b*). Participants were next asked to study 15 photographs of public greenspace without SuDS (electronic supplementary material, S2) for 2 min, and then completed the No-SuDS feeling thermometer. Finally, participants completed the IAT via tablet computer (§3*c*). One hundred and twenty-five residents were surveyed in each location. This resulted in a final sample of 110 respondents for NGP and 83 respondents for Benton, with both tests fully completed and valid.

Images rather than words were used as the target-concepts to reduce any potential bias introduced by a lack of lay understanding of SuDS terminology. All images used in the tests were of greenspace and SuDS at the neighbourhood level and in public space, likely implemented by the Local Authority, EA or Water Company. No individual-level interventions, such as garden soakaways or residential green roofs, were included, as earlier research has shown that BGI at different scales is ruled by different values [31]. Therefore, the perceptions identified in this study are limited to greenspace and SuDS in the public realm.

### (b) Explicit test: feeling thermometer

Participants completed six feeling thermometers to assess their feelings with regards to the safety, attractiveness and tidiness of public greenspace with and without SuDS (electronic supplementary material, S3). Participants were asked to draw an X on the respective scales to indicate how they felt. Scales ranged from 0 (extremely unsafe, unattractive or untidy) to 100 (extremely safe, attractive or tidy). Averages of the three scores for SuDS, and three scores for no-SuDS, were calculated. Thermometer difference (TD) scores were then calculated by subtracting the average SuDS score from the average No-SuDS score and then normalized to a −2 to +2 scale to be consistent with the IAT *D*-score. Positive TD-scores indicate a preference for public greenspace with SuDS (negative scores reflect a preference for public greenspace without SuDS).

### (c) Implicit association test

The IAT method described by Greenwald *et al.* [22], was followed, adapted to compare the automatic associations of public greenspace with and without SuDS, and built on a trial version of the IAT conducted in Bristol in 2018 [27]. The IAT was administered on a Linx 1010 Pro Tablet with an attached keyboard, using the FreeIAT software [32]. Two types of stimuli were used: target-concepts and evaluative attributes. Photographs of public greenspace with and without SuDS represented the target-concepts. These were the same 30 photographs that were shown to participants at the start of the study as examples of greenspace with and without SuDS. A set of 24 positive and 24 negative words represented the evaluative attributes (table 1). These words were originally selected from an online thesaurus as frequently used English language synonyms for positive and negative concepts. As IAT scores typically reflect attitudes towards the overarching target-concepts rather than attitudes towards the individual exemplars of those concepts [21], the selection of the words was of secondary importance; of primary importance was that the words were easy to visualize and unambiguously classifiable as positive or negative. The average word lengths in each category were closely matched; the average number of letters in the positive and negative word categories was 6.5 and 7.2, respectively.

**Table 1.** Positive and negative evaluative attribute words used in the Newcastle Implicit Association Tests (IATs).

positive evaluative attribute words	negative evaluative attribute words
accurate, assured, beautiful, clean, comfort, cosy, cushion, expert, fine, gentle, happy, likeable, maintained, neat, organized, pleasant, pleasing, pretty, protect, pure, shelter, smart, trim, welcome	awful, deformed, dirty, disagreeable, disorder, displease, fear, ghastly, haphazard, haywire, hideous, hurt, injury, litter, monster, perilous, scruffy, terrible, threat, treacherous, turmoil, unsightly, unstable, waste

Each IAT began with an introduction to the test and instructions to the participants, provided by the FreeIAT software. The IAT consists of five blocks (table 2). Each block contains 20 trials, and each trial is associated with one stimulus, either a photograph or an evaluative attribute word. Stimuli are randomly selected in all tests and then entered back into the selection processes, i.e. a photograph or attribute word could appear multiple times during one trial block. During the test, the randomly selected stimuli are presented, one at a time, in the centre of the tablet screen and participants are asked to categorize each stimulus as quickly as possible using the left (e) and right (i) keys. The categories that the ‘e’ and ‘i’ keys represent are listed at the top of the tablet screen and change over the course of the five blocks. This is illustrated in table 2, with the solid black circles indicating the allocation of the stimulus to either the left (e) or right (i) hand responses. For instance, in Block 1, the participant would select the ‘e’ key if the stimulus was a photograph of SuDS, or the ‘i’ key if the photograph was of greenspace without SuDS.

**Table 2.** Trial blocks in the Implicit Association Test (IAT). A solid black circle indicates allocation of a target or attribute to a left (e) or right (i) hand response. Modified after [22].

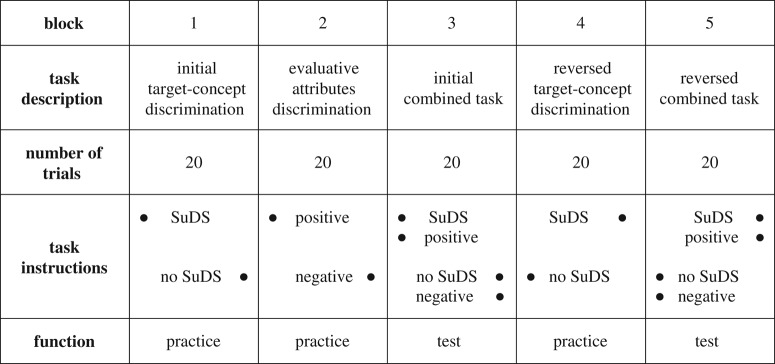

Each stimulus is shown on the screen until a correct response is registered. A correct response refers to the classification of the stimulus into the pre-selected categories. This refers to the positive or negative categories for the evaluative attribute words or the ‘SuDS’ or ‘No-SuDS’ categories for the images. Classifying the word ‘beautiful’ as negative, for example, would constitute an incorrect response, as would classifying an image of greenspace without SuDS as ‘SuDS’. If an incorrect response is given, a red ‘X’ appears on the screen and the respondent is required to select the correct response key for the test to continue.

If a participant differentially associates target-concepts with evaluative attributes then they should find one of the combined tasks (blocks 3 or 5) easier (or faster to respond to) than the other. This provides a measure of implicit attitudinal difference among the target-concept categories. The IAT effect (called the ‘difference’ or *D*-score) is the difference between the average response time across all trials in block 5 minus the average response time in block 3. *D*-scores were calculated using the improved scoring algorithm, adapted for five blocks rather than the original seven [33]. As part of the scoring algorithm trials with response times greater than 10 000 ms, or less than 300 ms for more than 10% of their trials, were first removed. The block mean of correct trials +600 ms was added to trials initially answered incorrectly. A high *D*-score indicates that public greenspace with SuDS was more closely associated with positive concepts, and/or less closely associated with negative concepts, than public greenspace without SuDS. *D*-scores between −0.2 and +0.2 are considered neutral, indicating no preference [24]. Implicit perceptions of attractiveness, tidiness and safety are not assessed directly but influence responses to the IAT.

## 4. Results

**Figure 3.**
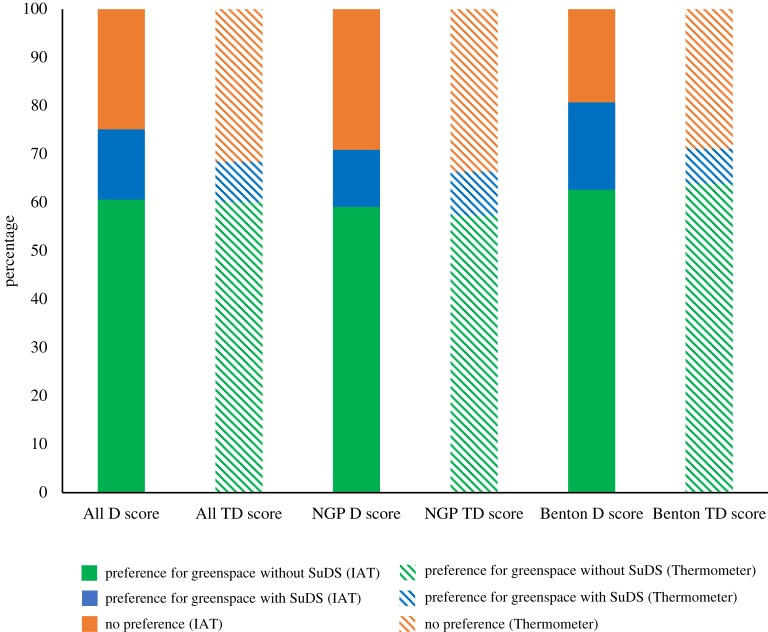
Illustrating the percentages of respondents who demonstrated preferences for greenspace without SuDS, greenspace with SuDS or no preference, for all Newcastle data (*n* = 193), Newcastle Great Park (NGP) (SuDS; *n* = 110) and Benton (greenspace, no-SuDS; *n* = 83), as determined by the Implicit Association Test (IAT) *D*-scores and Feeling Thermometer Difference (TD) scores. (Online version in colour.)

Explicit TD-scores ranged from −2.00 to 1.09 (mean = −0.53, s.d. = 0.70, *n* = 193), indicating that the sample population has an explicit preference for public greenspace without SuDS (table 3: all scores are provided in the electronic supplementary material, table S4). With regard to the individual respondents, 60% illustrated an explicit preference for public greenspace without SuDS compared with 32% who gave neutral responses and 8% who demonstrated a preference for SuDS (figure 3). Implicit *D*-scores ranged from −1.13 to 1.47 (mean = −0.26, s.d. = 0.45, *n* = 193), indicating a slightly weaker implicit preference for public greenspace without SuDS within the sample population; 61% of individual responses illustrated an implicit preference for public greenspace without SuDS. This was much higher than the 14% that demonstrated an implicit preference for SuDS or a neutral response (25%) (figure 3).

**Table 3.** Mean *D*-scores (IAT), TD-scores (feeling thermometer) and scores in the three feeling thermometer subcategories (attractiveness (attract.), safety and tidiness). All scores are normalized to a −2 to 2 scale, standard deviation is given in parentheses. The larger the score the greater preference for the target variable. NGP, Newcastle Great Park. Thermometer sub-category scores in the SuDS categories for all samples, NGP and Benton were significantly different to the corresponding scores in the no-SuDS categories (Mann–Whitney *U*-tests).

			SuDS			no-SuDS		
	*D*-score	TD-score	attract.	safety	tidiness	attract.	safety	tidiness
all (*n* = 193)	−0.26 (0.45)	−0.53 (0.70)	0.8 (0.8)	0.7 (0.7)	0.5 (0.9)	1.1 (0.5)	1.2 (0.4)	1.2 (0.4)
NGP (*n* = 110)	−0.27 (0.40)	−0.51 (0.74)	0.7 (0.8)	0.8 (0.8)	0.4 (0.9)	1.2 (0.4)	1.1 (0.4)	1.2 (0.4)
Benton (*n* = 83)	−0.26 (0.51)	−0.55 (0.64)	0.8 (0.7)	0.7 (0.7)	0.5 (0.8)	1.2 (0.4)	1.1 (0.5)	1.3 (0.4)

**Figure 4.**
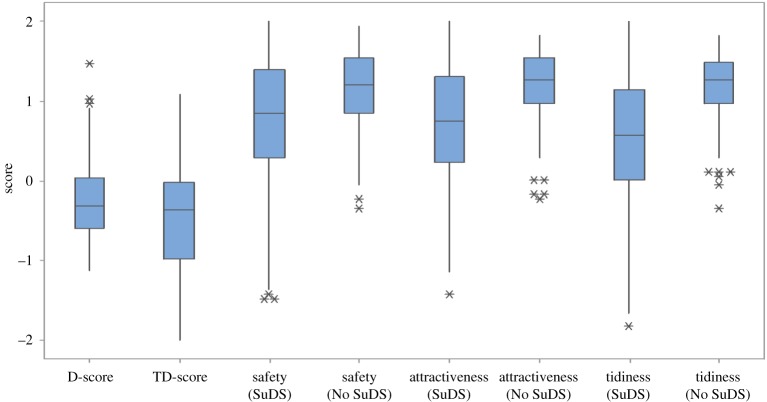
Distributions of *D*-scores (IAT) and normalized TD-scores (feeling thermometer), and feeling thermometer scores for SuDS and No-SuDS in the three categories (safety, attractiveness and tidiness). The median score is denoted by the centre line, the box denotes the interquartile range (IQR) and the upper (and lower) whiskers extend to the maximum (and minimum) data point within 1.5 times the IQR. Outliers (starred) are data points beyond the lower whiskers. (Online version in colour.)

A weak but statistically significant correlation was observed between TD-scores and *D*-scores (*r* = 0.26, *p* = 0.00), comparable with correlations reported between explicit tests and IATs in earlier research [19,34,35]. Despite this, TD-scores and *D*-scores were significantly different (*p*-value = 0.004, Mann–Whitney *U* test). This is thought to be due to the higher variability of TD-scores, when compared with *D*-scores, as illustrated by the higher density of negative responses, and greater interquartile range (IQR) and longer whiskers in the respective box plots (figure 4).

The positive average scores in the three feeling thermometer subcategories show that respondents have positive feelings towards both greenspace with and without SuDS (table 3), regarding these spaces as attractive, tidy and safe. By comparison, greenspace without SuDS is regarded as safer, more attractive and tidier than greenspace with SuDS (figure 4, table 3). This was observed for the whole dataset, as well as NGP and Benton sub-datasets. The smaller standard deviations and IQRs for scores representing safety, attractiveness and tidiness of greenspace without SuDS suggest greater agreement within the sample population. The negative whiskers in the SuDS feeling thermometers (extending to the minimum data point within 1.5 times the IQR, figure 4), and the outliers (classified as any data points beyond the lower whisker) consequentially reduce the mean and suggest that some respondents regard SuDS as unattractive, unsafe and untidy. Nonetheless, this represents a minority of strong negative preferences within the general population who regard SuDS more favourably. Tidiness of SuDS, in particular, received the lowest mean score when compared with attractiveness and safety (all samples, NGP and Benton sub-datasets, table 3). Out of the three scores that respondents gave in the SuDS thermometers, only 13% gave their highest score to the thermometer evaluating tidiness, compared with 34% who scored attractiveness the highest, and 22% who scored safety the highest (the remaining 31% of respondents gave the same highest score in two thermometers). A significant positive correlation was observed between SuDS attractiveness and tidiness (*r* = 0.866, *p* = 0.000), and attractiveness and safety (*r* = 0.671, *p* = 0.000).

Comparison of preferences recorded in NGP (proximity to SuDS) and Benton (proximity to greenspace) revealed no significant differences between implicit *D*-scores (*p* = 0.831, Mann–Whitney *U* test) or explicit TD-scores (*p* = 0.502, Mann Whitney *U* test). Implicit and explicit preferences for public greenspace without SuDS were observed, mirroring the trends of the whole sample population (figure 3, table 3). Overall explicit preferences for greenspace without SuDS were slightly stronger than implicit preferences; mean TD-scores were −0.51 and −0.55 for NGP and Benton, respectively, compared with mean *D*-scores of −0.27 and −0.26 (NGP and Benton, respectively).

## 5. Discussion

The consolidation of implicit and explicit tests that we present contributes to our growing understanding of the complexity of public perceptions and myriad factors that influence people's attitudes and preferences, which affects their subsequent behaviour. To our knowledge, we present the first complete study of the implicit perceptions of SuDS, building on our trial conducted in Bristol [27], and the first comparative quantification of explicit perceptions of attractiveness, safety and tidiness of greenspace with and without SuDS, as examples of characteristics that residents frequently use to assess the value of these spaces [7,14].

### (a) Explicit and implicit preferences for greenspace with or without SuDS

The majority of respondents in the sample population positively regard greenspace with and without SuDS, suggesting that both types of public greenspace are valued components of landscapes and developments. Nonetheless, respondents implicitly and explicitly have a slight preference for greenspace without SuDS and perceive greenspace without SuDS as more attractive, tidier and safer. The stronger explicit (compared with implicit) preferences that were exhibited overall may be because respondents have a stronger conscious opinion on the use of greenspace and actively differentiate between the benefits provided by greenspace with SuDS and greenspace without SuDS, compared with a subconscious that does not have a strong internalized concept of SuDS and feels more neutral about this use of space.

We assumed that social desirability bias would increase the positive scores given to greenspace with SuDS in the explicit tests as part of an embedded response of ‘liking’ all greenspace. The smaller percentage of respondents explicitly favouring SuDS (8%), compared with the higher percentage of respondents implicitly favouring SuDS (14%), suggests that social desirability bias does not favour SuDS. Respondents may report more negative and neutral views in the explicit tests because they are rationalizing about the advantages and disadvantages associated with SuDS, e.g. attractiveness versus safety, and consciously decide to highlight socially acceptable concerns such as tidiness and safety. By contrast, there may be some social bias in the explicit tests with respondents expressing more concern about tidiness and safety than they instinctively feel, as illustrated by the negative tails on the SuDS feeling thermometers when compared with the fewer negative (implicit) *D*-scores (figure 4). This may imply that safety and tidiness are important features of SuDS but that designers should take care not to put too much weight on a minority of strong preferences.

The range of *D*-scores (−1.13 to 1.47) suggests that some within the sample population do have stronger attitudes towards the use and appearance of greenspace. The higher variability of the explicit TD-scores further suggests that the sample population does not conform to a common preference, especially when examined by explicit measures. This is further illustrated by the wide range of scores for the individual feeling thermometers measuring perceived safety, attractiveness and tidiness of greenspace with SuDS (figure 4), and in particular, the presence of several highly negative scores for tidiness (the lowest score was −1.83, compared with −1.49 for both attractiveness and safety).

#### (i) Perceptions of attractiveness, tidiness and safety

Overall, there was consistency in how respondents explicitly rate attractiveness, safety and tidiness, as represented by the significant positive correlation between thermometer scores. This is a key finding and contrasts with earlier literature that reports an aesthetic value associated with SuDS yet a dislike of SuDS due to concerns over health and safety risks and litter [5,6,13,14]. Generally, respondents who regarded SuDS as attractive also regarded them as safe and tidy (and vice versa), suggesting that people's conscious views on SuDS are more consistent than they have previously appeared in other studies.

Nonetheless, SuDS were not unanimously regarded as attractive, and for many respondents, the aesthetic value of greenspace was scored more highly, illustrated by the lower mean score for attractiveness in the SuDS feeling thermometer and a larger number of negative scores (compared with the mean attractiveness score for no-SuDS, figure 4). This may be explained by several factors: a genuine preference for the aesthetics provided by greenspace; a dislike of some of the images presented in the tests that contain SuDS; and/or previous interaction with SuDS that the respondents did not regard as aesthetically pleasing; 60% of the SuDS images illustrated open water (SuDS ponds or retention basins) and most images contained reed beds. The visual properties of ‘blue space’ are known to be explicitly viewed as attractive [36] hence the choice of vegetation may exert a greater influence on overarching preferences. SuDS are seen as ‘messy’, particularly if certain plant species are used (e.g. *Junctus* rushes) that may be mistaken for overgrown grasses and weeds [5]. Environments with ‘tidier’ nature, as portrayed in the images of public greenspace without SuDS used in this study that comprise short, well-maintained grass and mature trees, may be more aesthetically pleasing. As SuDS were, on average, regarded as attractive, we can infer that ‘tidy SuDS’ would be regarded as even more attractive. The positive correlations between SuDS safety, and tidiness, with attractiveness suggest that public perceptions of SuDS aesthetics could be improved by the presence of ‘tidier’ vegetation, perhaps adjacent to detention basins or swales, and less visible open water, reeds and rushes. This may, however, reduce the potential for the SuDS to improve water quality, which is a key function of reedbeds, thus requiring a trade-off for the provision of different co-benefits. Nonetheless, further investigation into specific types of SuDS and greenspace that are ranked highly for attractiveness, safety and tidiness is an essential line of future research if SuDS are to be designed for maximum acceptance by local residents.

### (b) The influence of proximity to SuDS on preferences

The lack of significant differences between both implicit and explicit preferences recorded in NGP and Benton showed that residential proximity to SuDS did not affect the response to the IAT or feeling thermometers, implying a lack of influence of proximity. This contrasts with previous findings in NGP which found that understanding of SuDS functionality was linked to positive perceptions and willingness to engage [30]. It is possible that the responses of NGP residents reflect perceptions of SuDS in general and were based on the images of SuDS shown at the start of the test, only one of which was of an NGP SuDS feature. We used images rather than words to reduce any potential bias introduced by a lack of lay understanding of SuDS terminology; however, using words would have required participants to rely on their internal understanding of the SuDS concept to complete the tests, which would have resulted in responses that were more dependent on experience, and knowledge and awareness of SuDS. Further investigation of the level of representation of the stimuli would benefit future IATs in this research area. In reality, the NGP residents may value the greenspace provided by the NGP SuDS without acknowledging that they are sustainable drainage features.

The fact that preferences for public greenspace were not influenced primarily by residential location in our sample may be inherently linked to how the respondents value different benefits provided by blue and green infrastructure, e.g. amenity, biodiversity, flood and water management, or recreation, and represents an avenue of further research. Other attributes of SuDS and greenspace that were not covered in the explicit tests may also influence overarching perceptions, e.g. knowledge of SuDS functionality, the respondent's environmental attitudes more generally, e.g. around climate change or presence of nature in cities, or past experience with flooding, as implicit attitudes, as well as explicit, can also be altered as a result of a powerful affective experience [35].

### (c) Directions for future research

Behaviour has been shown to be best predicted by a combination of implicit and explicit attitudes [36]. Determining what drives people's behaviours around greenspace and SuDS, and the roles that both conscious (explicit) and unconscious (implicit) awareness play, were beyond the scope of our study but present an interesting focus for future research. Implicit attitudes are more predictive for spontaneous behaviour, whereas explicit attitudes are more predictive for deliberate behaviour [37], and hence, it is possible that implicit attitudes affect some types of behaviours more than others. For example, Perugini [37] found that implicit attitudes predicted rapid choice about whether test respondents take a free snack or a piece of fruit on the spot (an example of spontaneous behaviour). Implicit attitudes also direct much of people's automatic behaviours and could be strengthened to encourage specific behaviours. For instance, building on the implicit association between women and nature, based on the ‘Mother Nature’ association in mental imagery, could increase pro-environmental behaviour and help people develop their desire to protect the environment [26]. Future research to investigate the potential relationship between implicit associations of greenspace and SuDS, and behaviours of users in these spaces, would advance our knowledge of how implicit perceptions may drive certain types of behaviour. To more fully understand any disconnect between expressed positive attitudes to greenspace and behaviours around them, implicit and explicit perceptions studies could be combined with measures to determine levels of pro-environmental behaviour, such as monitoring or maintenance as part of local stewardship activities.

In the geographically targeted investigation presented in this paper, we evaluated whether perceptions differed if respondents lived in proximity to SuDS or greenspace without SuDS. Perceptions, attitudes and resulting behaviour may also be influenced by many other factors including: broader environmental attitudes, e.g. around climate change or water conservation [17]; awareness of purpose, function and perceptions of risk [5]; past experiences with flooding, especially if a flood event happened after the installation of a SuDS asset; the age of the SuDS features; the level of maintenance; how accepted the SuDS are as a part of the landscape (how ‘used to it’ residents are) and demographic factors such as residents' qualifications, ethnic group or levels of unemployment. Where and how facilities are positioned within the public realm, and how they are used, e.g. for social activities, exercise, recreation, relaxing or dog walking, will further influence behaviours in these spaces and may provide greater insight into behaviours than simple demographics [15]. By using the IAT as a tool to measure implicit associations, future research can explore a number of applied questions. This could include investigating the impact of flood events on implicit perceptions of SuDS and other types of flood risk management infrastructure as direct experience with extreme weather events has been shown to be an effective catalyst for changing implicit attitudes [35].

## 6. Conclusion

Understanding the perceptions of different types of blue–green space in the public realm, e.g. SuDS and BGI, is a fundamental step to addressing the socio-political barriers to their implementation, improving awareness of functionality and delivery of multiple co-benefits and gaining public support. The novel image-based application of the IAT that is presented investigates unconscious perceptions of SuDS in public greenspace. Combining the IAT with the explicit feeling thermometers provides complementary information on explicit preferences of greenspace with and without SuDS and further evaluates attitudes towards attractiveness, safety and tidiness to provide the first comparative quantification, to date, of these perceptions.

The IAT contributes additional insight that cannot be captured by explicit tests that suffer from social desirability bias and can uncover hidden perceptions that are more entrenched in each respondent's value system. Greenspace with and without SuDS is perceived positively by the sample population in Newcastle, suggesting that they are valued components of landscapes and developments. Overall, greenspace without SuDS was implicitly and explicitly preferred, and greenspace without SuDS regarded as more attractive, tidier and safer. However, the wide distribution of implicit and explicit tests scores for greenspace with SuDS illustrates the complexity of public perceptions of blue–green space. The fact that the strongly negative explicit scores were not reflected in the implicit scores may suggest that explicit attitudes towards tidiness and safety may not be deep-rooted and may be subject to some social bias influenced by, for example, concerns around public space that are deemed to be more socially acceptable. For instance, the data suggest that a small proportion of respondents are more concerned about tidiness and safety than they instinctively feel, perhaps because they are rationalizing about the advantages and disadvantages associated with SuDS and consciously decide to highlight socially acceptable concerns, such as tidiness and safety. Although safety and tidiness are important features of SuDS, designers should take care not to put too much weight on strongly negative preferences expressed by a minority. Residential proximity to SuDS, which was investigated by comparing responses from residents living in properties within 500 m of the NGP SuDS, or near to large areas of recreational greenspace in Benton, did not significantly influence test scores. Several factors may instead influence perceptions, including respondents' broader environmental attitudes, e.g. around climate change or water conservation, past experiences with flooding or knowledge of SuDS functionality, and requires further investigation.

The value of combining implicit and explicit tests lies is the ability to overcome potential biases associated with explicit tests alone and improve the understanding of any disconnect between expressed positive attitudes to natural spaces and behaviours around them. Implicit perceptions, attitudes and preferences have been little studied in the flood and water management discipline yet may play a pivotal role in influencing overarching attitudes towards blue–green space. Further investigation of implicit preferences is recommended to establish what may be driving attitudes towards SuDS and BGI and inform infrastructure design to promote the aspects that people more highly value in order to create blue–green space that is desired, appreciated and supported by local residents.

## Supplementary Material

Supplementary Material 1

## Supplementary Material

Supplementary Material 2

## Supplementary Material

Supplementary Material 3

## Supplementary Material

Supplementary Material 4

## Data accessibility

The data associated with this publication are provided in the electronic supplementary material.
