# Supplementary material for: Developing the implicit association test to uncover hidden preferences for sustainable drainage systems
Source: Philos Trans A Math Phys Eng Sci. 2020 Feb 17;378(2168):20190207. doi: 10.1098/rsta.2019.0207 (PMC7061966; doi:10.1098/rsta.2019.0207)

Supplementary material for O'Donnell et al., 2020, Developing the Implicit Association Test to uncover hidden preferences for sustainable drainage systems, *Phil. Trans. R. Soc. A.* DOI:10.1098/rsta.2019.0207.

Supplementary Material 1: Images of public greenspace that contain SuDS (sustainable drainage systems) that were shown to respondents before they completed the feeling thermometers and IAT (Implicit Association Test). These images were also used as target-concepts (SuDS) in the IATs.

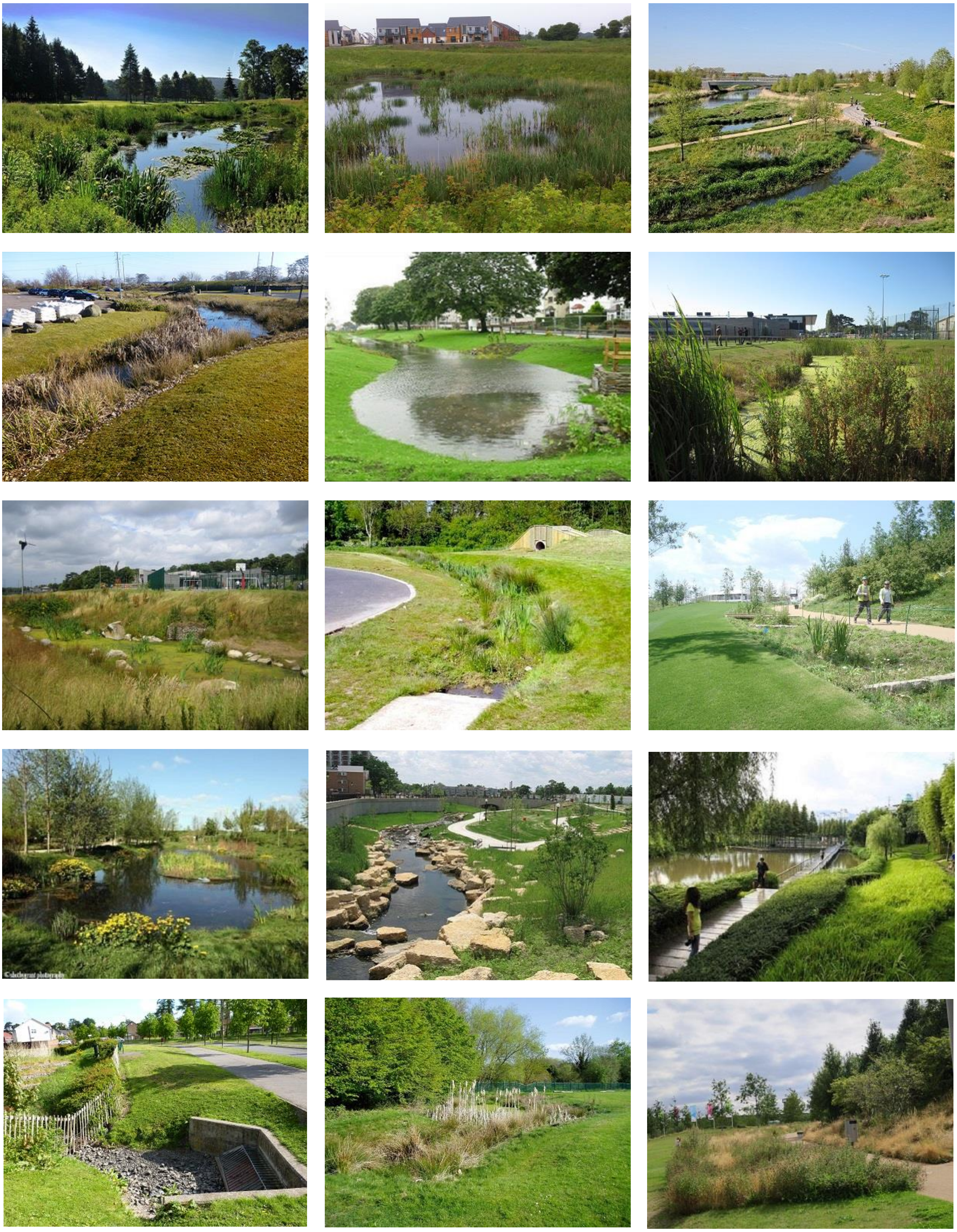

Supplement: Supplementary Material 1 [file rsta20190207supp1.pdf]
