# Supplementary material for: Developing the implicit association test to uncover hidden preferences for sustainable drainage systems
Source: Philos Trans A Math Phys Eng Sci. 2020 Feb 17;378(2168):20190207. doi: 10.1098/rsta.2019.0207 (PMC7061966; doi:10.1098/rsta.2019.0207)

Supplementary material for O’Donnell et al., 2020, Developing the Implicit Association Test to uncover hidden preferences for sustainable drainage systems, *Phil. Trans. R. Soc. A.* DOI:10.1098/rsta.2019.0207.

Supplementary Material 2: Images of public greenspace that do not contain SuDS that were shown to respondents before they completed the feeling thermometers and IAT. These images were also used as target-concepts (no-SuDS) in the IATs.

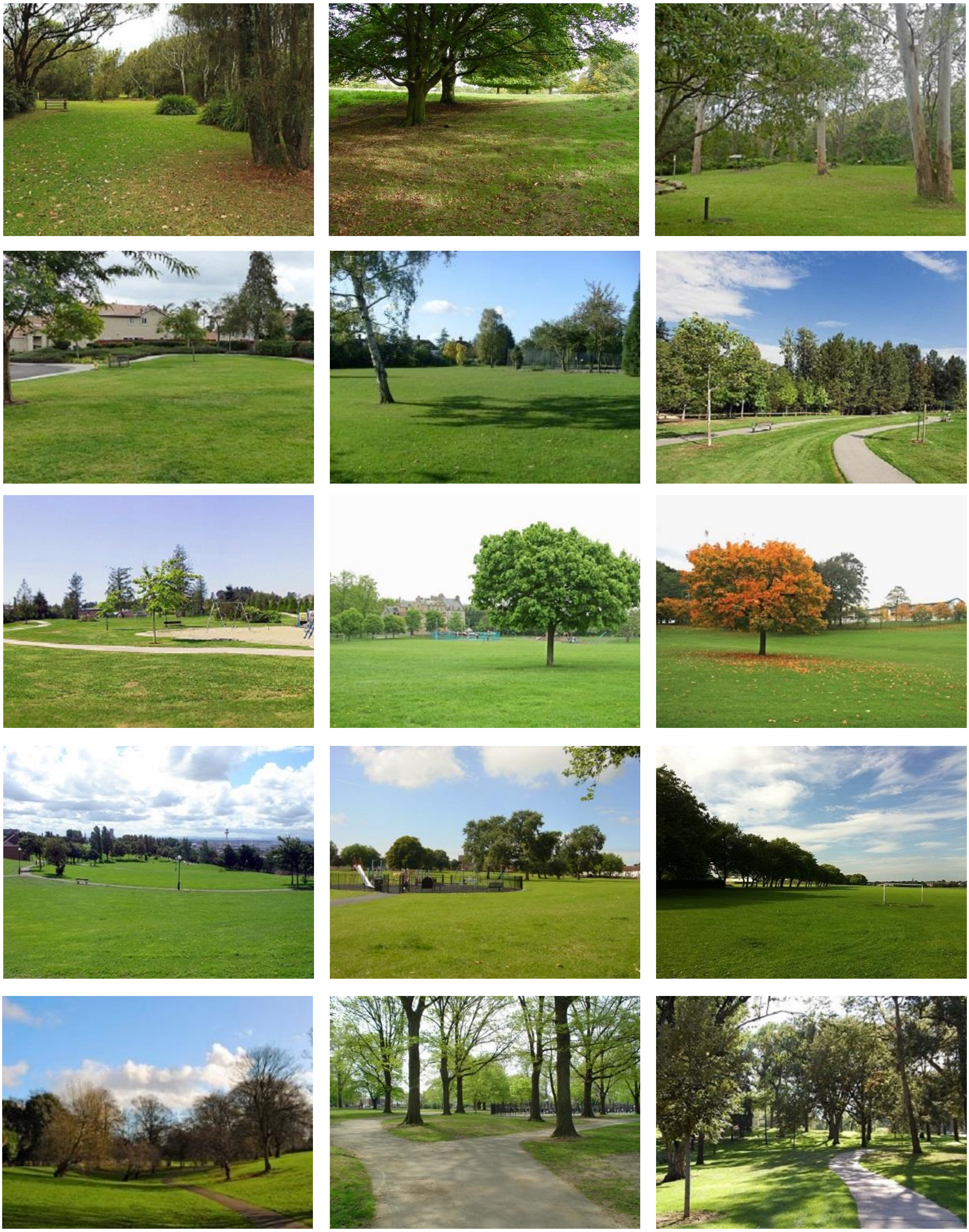

Supplement: Supplementary Material 2 [file rsta20190207supp2.pdf]
