# Supplementary material for: Developing the implicit association test to uncover hidden preferences for sustainable drainage systems
Source: Philos Trans A Math Phys Eng Sci. 2020 Feb 17;378(2168):20190207. doi: 10.1098/rsta.2019.0207 (PMC7061966; doi:10.1098/rsta.2019.0207)
Supplement: Supplementary Material 3 [file rsta20190207supp3.pdf]

Supplementary material for O'Donnell et al., 2020, Developing the Implicit Association Test to uncover hidden preferences for sustainable drainage systems, *Phil. Trans. R. Soc. A.* DOI:10.1098/rsta.2019.0207.

Supplementary Material 3: Feeling thermometers investigating how safe, attractive and tidy respondents believe public open greenspace with and without SuDS to be.

100 Extremely **safe**

*How **safe** do you feel these public open spaces are?*

Draw an X on the scale to indicate how you feel.

0 Extremely **unsafe**

100 Extremely **attractive**

*How **attractive** do you feel these public open spaces are?*

Draw an X on the scale to indicate how you feel.

0 Extremely **unattractive**

100 Extremely **tidy**

*How **tidy** do you feel these public open spaces are?*

Draw an X on the scale to indicate how you feel.

0 Extremely **untidy**
